# Genome-Wide Analysis of Genetic Diversity in *Plasmodium falciparum* Isolates From China–Myanmar Border

**DOI:** 10.3389/fgene.2019.01065

**Published:** 2019-10-29

**Authors:** Run Ye, Yini Tian, Yufu Huang, Yilong Zhang, Jian Wang, Xiaodong Sun, Hongning Zhou, Dongmei Zhang, Weiqing Pan

**Affiliations:** ^1^Department of Tropical Diseases, Naval Medical University, Shanghai, China; ^2^Yunnan Institute of Parasitic Diseases, Puer, China

**Keywords:** *Plasmodium falciparum*, genomes, China–Myanmar border, diversity, genetic marker

## Abstract

*Plasmodium falciparum* isolates from China–Myanmar border (CMB) have experienced regional special selective pressures and adaptive evolution. However, the genomes of *P. falciparum* isolates from this region to date are poorly characterized. Herein, we performed whole-genome sequencing of 34 *P. falciparum* isolates from CMB and a series of genome-wide sequence analyses to reveal their genetic diversity, population structures, and comparisons with the isolates from other epidemic regions (Thai–Cambodia border, Thai–Myanmar border, and West Africa). Totally 59,720 high-quality single-nucleotide polymorphisms (SNPs) were identified in the *P. falciparum* isolates from CMB, with average nucleotide diversity (π = 4.59 × 10^−4^) and LD decay (132 bp). The Tajima’s *D* and Fu and Li’s *D* values of the CMB isolates were −0.8 (*p* < 0.05) and −0.84 (*p* < 0.05), respectively, suggesting a demographic history of recent population expansion or purifying selection. Moreover, 78 genes of the parasite were identified that could be under positive selection, including those genes conferring drug resistance such as *pfubp1*. In addition, 33 SNPs were identified for tracing the source of the parasites with a high accuracy by analysis of the most differential SNPs among the four epidemic regions. Collectively, our data demonstrated high diversity of the CMB isolates’ genomes forming a distinct population, and the identification of 33-SNP barcode provides a valuable surveillance of parasite migration among the regions.

## Introduction

The global malaria burden caused by *Plasmodium falciparum* has been substantially reduced since the artemisinin-based combination therapies were recommended as first-line antimalarial drugs for treatment of *falciparum* malaria in all the endemic regions. According to the World malaria report of WHO, however, there were an estimated 219 million cases and 435,000 malaria-related deaths in 2017. Majority of the malaria cases occurred in the countries in sub-Saharan Africa and India, and children aged under 5 years accounted for 61% of the global malaria deaths (WHO, 2018). However, malaria is still highly prevalent in Southeast Asia. In particular, the Greater Mekong Subregion in this area is an epicenter for emerging resistance of parasite to several antimalarial drugs, choloroquine and pyrimethamine and recently artemisinin (Eyles et al., 1963; Hurwitz et al., 1981; Dondorp et al., 2009). In China, since the national malaria elimination program was launched in 2010, local transmission of malaria has been effectively controlled according to the malaria report of China (Zhang et al., 2017), and no local malaria infection was reported in 2017, although of over 2,000 of the imported malaria cases. However, in the surrounding countries such as Myanmar, malaria burden is still high. In the past years, the number of imported malaria cases has been continuously increasing in China due to the increasing international exchange activities, especially in the border regions. In the malaria-free areas, tracing the origins of new malaria infections (local or imported) is pivotal to the maintenance of malaria-free zones and prompt responses to the individual cases, interrupting the parasites transmission cycle, and, ultimately, to disease eradication. However, the development of such tools has received little attention to date except in the study of drug resistance. Analysis of genetic diversity and population structure of *P. falciparum* should provide a fundamental basis for developing tools to trace the origins of new malaria infections in malaria-free zones.

With rapid development of sequencing technologies, a large number of *P. falciparum* isolates worldwide have been sequenced and have generated the parasite genomic data that are shared by large collaborative initiatives such as the MalariaGEN *P. falciparum* Community Project and the Pf3k Consortium. Analysis of the whole genome variation has generated numerous high-density single-nucleotide polymorphisms (SNPs) of the parasite. Application of the genomic approaches has identified new parasite genes that conferred the resistance to antimalarial drugs such as *k13*, *plasmepsin 2-3* (Ariey et al., 2014; Amato et al., 2017; Witkowski et al., 2017). However, the genomes of *P. falciparum* isolates from Southern China to date are poorly characterized, which could hinder the joint research with those in other endemic areas in the world. Therefore, in this study, we performed a deep sequencing of whole genome of 34 *P. falciparum* isolates from China–Myanmar border (CMB). We aim to reveal the genetic diversity and population characteristics of the parasite in this region. Moreover, we determined that their genetic compositions are reflective of their geographical origin through comparison of the genome data of isolates from four other endemic areas.

## Materials and Methods

### Sample Collection and Processing

The isolates were collected from symptomatic malaria patients diagnosed by microscopic examination from 2007 to 2010. The 43 isolates of *P. falciparum* were collected from a valley where there are about 50,000 residents. This collecting site is located on the CMB (longitude: 97°33′47.81″; latitude: 24°45′21.65″). There is a stream through the valley, named “Nabang” on the China side and “Lazan” on the Myanmar side (named the “Nabang–Lazan valley”). The eight isolates of *P. falciparum* were collected from the townships of Trat and Srisaket along the Thai–Cambodia border (TCB) in 2010; two isolates of *P. falciparum* were collected from returned Chinese migrant workers from Nigeria in 2016. The samples were collected as part of research. Short-term (2–4 weeks) cultivation of the parasite isolates was established *in vitro* to get enough DNA of parasites in O^+^ human red blood cells in complete RPMI 1640 medium under an atmosphere of 5% O_2_, 5% CO_2_, and 90% N_2_. Genomic DNA of the parasites was extracted using the QIAamp DNA Blood Mini Kit (Qiagen). Samples were sequenced on the Illumina HiSeq 2000 or HiSeq x-ten platforms according to the manufacturer’s protocol (Bentley et al., 2008), with a minimum of 150 bp (or 100 bp) paired-end reads. The Illumina sequencing reads have been submitted to European Nucleotide Archive (ENA); accession numbers are provided in **Supplementary Table 1**. This study has been approved by the internal review board of Naval Medical University in China, and written informed consent was obtained from the patients.

### Additional Data Sets

The raw high-quality reads of the genomic data of parasite isolates from the other three endemic regions (e.g., TCB, Pursat and Pailin; TMB: Thai–Myanmar border, Mae Sot; WAF: West Africa, Banjul of Gambia and Navrongo of Ghana) (Miotto et al., 2013; Miotto et al., 2015) were downloaded from ENA (http://www.ebi.ac.uk/ena/) (**Supplementary Table 2**). The criteria for the selection of the comparator sequences of the isolates from ENA include >1.0 G of sequencing data per isolate and genome average coverage >30-fold.

### SNP Discovery and Quality Filtering

High-quality short reads were mapped on the 3D7 reference genome (version 8.1), using Burrows–Wheeler aligner (Li and Durbin, 2009) (http://bio-bwa.sourceforge.net) with default parameter and processed the resulting alignments using SAMtools (Li et al., 2009) and Picard v1.66 (Hupalo et al., 2016). Samples classified as mixed infections estimated by estMOI software (Assefa et al., 2014) and samples with average coverage <30-fold or <70% sequences mapping over 3D7 reference genome sequence were removed. GATK UnifiedGenotyper (McKenna et al., 2010) was used for high-quality SNP calling with the following criteria: (a) biallelic; (b) call rate >95%; (c) Quality scores of >30; (d) base quality >30; (e) sample average coverage, > 10-fold or <2000-fold; (f) not located in subtelomeric regions, the hypervariable *var*, *stevor*, and *rifin* gene families (**Supplementary Table 3**) (Ocholla et al., 2014). Heterozygous calls were converted to the majority genotype if the coverage ratio is ≥75%; otherwise, the alleles were coded as missing; sites with <5-fold coverage in a given sample were filtered out at this stage, and SNPs with call rates below 95% were also removed. The high-quality SNP data then were imputed by using BEAGLE v3.3.2 with default parameters (Browning and Browning, 2009)

### Genetic Diversity and Tajima’s *D*

Nucleotide diversity (π) was calculated with 5-kb sliding windows using VCF tools, Tajima’s *D* and Fu and Li’s *D* values were calculated using PopGenome package of R with default parameters (Pfeifer et al., 2014). Linkage disequilibrium (LD) was measured using PopLDdecay (minor allele frequency [MAF] >0.02) (https://github.com/BGI-shenzhen/PopLDdecay), and LD map was constructed for four groups based on the *r* ^2^ metric.

### Positive Selection

Signatures of positive directional selection were identified using the standardized |iHS| (integrated haplotype score); cross-population extended-haplotype homozygosity (XP-EHH) analyses were used to identify evidence for positive selection that have reached or are near fixation in one population but remain polymorphic in others. Both iHS and XP-EHH were calculated using the R package rehh with default parameters (Gautier and Vitalis, 2012).

### Population Structure

The Wright’s fixation index *F* *_ST_*, a measure of population differentiation due to genetic structure, was calculated using PopGenome package of R with default parameters (Pfeifer et al., 2014). Principal component analysis (PCA) was undertaken *via* R, and the neighbor-joining tree was constructed using MEGA6.0. We performed admixture analysis using ADMIXTURE software (Alexander and Lange, 2011), SNP pairs that appeared to be linked were excluded, and the optimal number of cluster was determined by performing multiple runs of the software under different K values (2–10). Graphs were created by ggplot2 (https://cran.r-project.org/web/packages/ggplot2/). Common SNPs (MAF > 0.02) were used in these analyses.

### Population Discriminant Model

SNP barcode was constructed through Stepwise Discriminant Analysis by SPSS 19.0 software. The most differential neutral SNPs (intergenic, intronic, or 4-fold degenerate sites) were chosen after the pairwise comparison of each of two populations by Plink (http://www.cog-genomics.org/plink2/) and then performed with the Stepwise Discriminant Analysis. Finally, a 33-SNP barcode was designed to classify all 163 samples from four different regions. In total, 51 isolates were used for the accuracy test of 33-SNP barcode, including 30 samples downloaded from ENA (TCB, n = 10; TMB, n = 10; WAF, n = 10); the remaining 21 were sequenced as described above (10 from CMB, one isolate from Hainan province of China, eight isolates from Trat and Srisaket of Thailand, two isolates from Nigeria) (**Supplementary Table 4**).

## Results

### Genetic Diversity of the Isolates From China–Myanmar Border

To investigate the parasite population feature, 43 *P. falciparum* isolates from Nabang–Lazan valley in CMB were collected and established for their *in vitro* continuous cultivation. The cultured parasite isolates were used for their genome sequencing. The sequence reads of the isolates were first aligned on the 3D7 reference genome sequence. Three isolates with less than 70% mapping over the reference and six isolates estimated as mixed infections by estMOI software were excluded for further analysis. The genome sequence data of the remaining 34 isolates were analyzed to identify putative SNPs using GATK. A series of quality control measurements (see *Materials and Methods*) was performed to remove high-risk genotypic-error SNPs. We identified a total of 59,720 high-quality SNPs from the CMB isolates, representing a density of one SNP every 390 bp. Of the SNPs, 67.09% can be mapped to the coding sequences. The allele frequency spectrum showed that majority of SNPs had a low MAF (<0.05) (**Supplementary Figure 1**). A 5-kb sliding-window approach was applied to calculate the nucleotide diversity (π) using VCF tools, and the average π of the isolates in this region was 4.59 × 10^−4^.

### Signatures of Selection in the Isolates From China–Myanmar Border

We then investigated any signature of selection of the parasite in this region. We found that Tajima’s *D* and Fu and Li’s *D* values of the CMB isolates were −0.8 (*p* < 0.05) and −0.84 (*p* < 0.05) across the entire genome, respectively, indicating a demographic history of recent population expansion or purifying selection. With the gene transfer format file containing information about gene structure, we annotated genes and then calculated Tajima’s *D* for the individual genes by R scripts. We found 3,858 genes had at least five SNPs, of which 158 genes had Tajima *D* values >1 (**Supplementary Table 5**), suggesting that these 158 genes are most likely under balancing selection. These genes include *ama1*, *msp1*, *eba175*, *dblmsp*, and *clag2*, which were reported previously for the balancing selection (Amambua-Ngwa et al., 2012).

**Figure 1:**
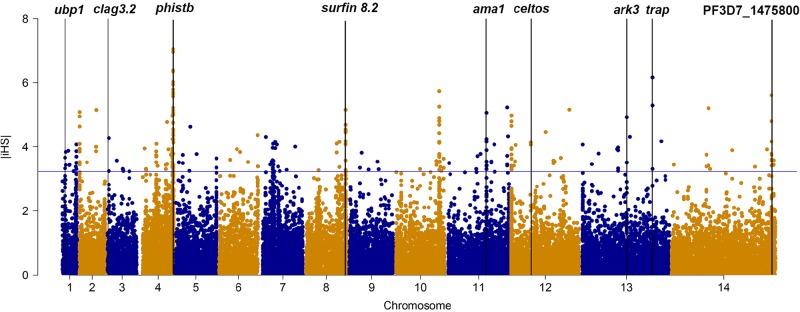
The top |iHS| hits in isolates from China–Myanmar border. *X* axis is chromosomes 1 to 14 in alternating colors; *y* axis is the value of |iHS|. The loci above the blue horizontal lines have the top 1% of |iHS| values.

We applied iHS to investigate genome-wide evidence for positive selection due to drug pressure or other mechanism (**Figure 1**). Using an iHS score of 3.23 (Top 1% expected distribution) as a strong hits threshold, we identified 78 genes that could be under significant positive selection, of which 32 genes had at least two SNPs (**Supplementary Table 6**). This analysis identified the selection signals of vaccine candidates genes (*ama-1*, *trap*, and *celtos*) and drug-resistant protein *pfubp1*, serine/threonine protein kinase *pfark3*, and so on. However, this analysis failed to detect selection signals of drug-resistant genes such as *pfcrt*, *pfdhfr*, *and pfdhps*. The reason could be that iHS may not be suitable for detecting positive selection for those SNPs that have reached fixation in local population.

### Comparison of Genetic Diversity of the Isolates Among Different Endemic Regions Worldwide

**Figure 2:**
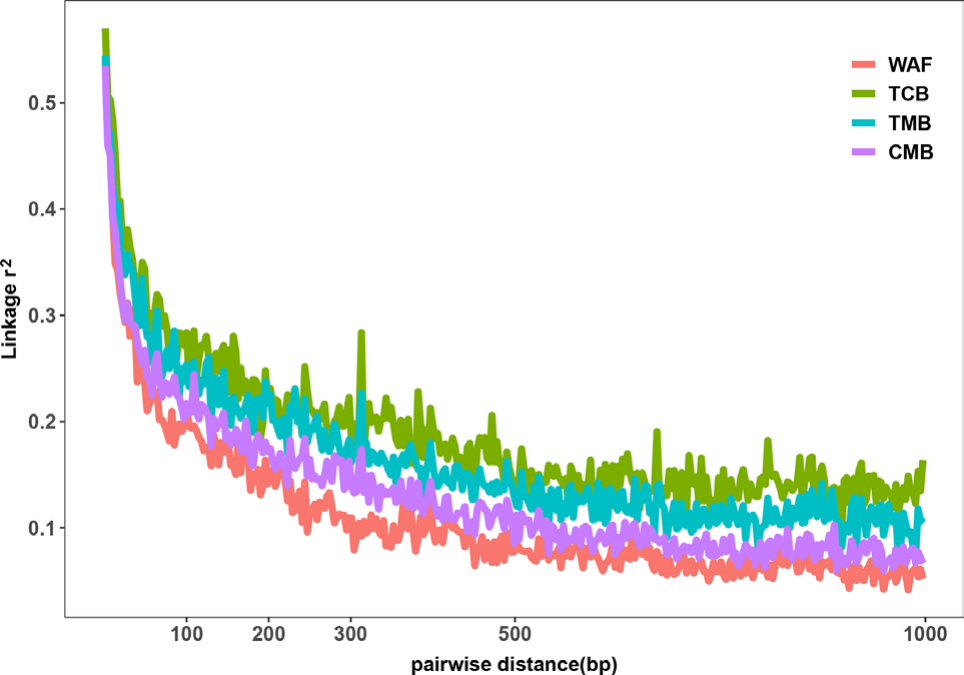
Linkage disequilibrium (LD) decay in four regions. LD decay was measured by *r* ^2^ between pairs of markers with MAF >2% and within 1,000 bp.

The outcomes from the above analysis showed high level of diversity in the genome sequence of the isolates from the CMB, generating over 50,000 of high-quality SNPs. We next investigated the genetic diversity among the *P. falciparum* isolates from different regions worldwide, including WAF (n = 40), TCB (n = 60), and TMB (n = 40) through obtaining their genome sequence data from ENA. Four isolates from TCB and seven isolates from WAF were excluded due to the estimated mixed infections or less than 70% mapping over the reference. We aligned sequence reads of the isolates on the 3D7 reference genome and applied GATK to identify putative SNPs. The average coverage of our dataset were 55x, which has no significant difference from that of publicly available dataset, including TCB(68x), TMB(74x) (p > 0.05). Thus, we characterized 150,761 SNPs passing the high-quality filters in 163 samples (CMB, n = 34; TCB, n = 56; TMB, n = 40; WAF, n = 33); a total of 90,669, 39,196, and 42,264 SNPs were generated from the isolate samples of WAF, TCB, and TMB, respectively (**Supplementary Figure 2**). Overall, there were 11,849 SNPs (7.9%) shared by all the isolates from the four regions. The parasites from CMB and TMB shared the largest number of SNPs (n = 28,310), while the isolates from WAF have the largest number of private SNPs [66,322 (44% of the total)], followed by the CMB isolates [21,662 (14.4% of the total)]. We calculated the nucleotide diversity of WAF (π = 5.20 × 10^−4^), TCB (π = 3.51 × 10^−4^), and TMB (π = 3.91 × 10^−4^) with 5-kb sliding windows, and permutation was performed to test whether there was significant difference in the nucleotide diversity among the four populations. The results showed that nucleotide diversity of CMB is significantly lower than WAF (Welsh *t* statistic = 4.7681, *p* < 2.2e−16), but higher than TCB (Welsh *t* statistic = −9.07, *p* < 2.2e−16) and TMB (Welsh *t* statistic = −5.44, *p* < 2.2e−16). The LD (*r* ^2^) was measured to examine the potential effects of recombination. Using common SNPs (MAF > 0.02), we found the LD level decayed to *r* ^2^ = 0.2 within 132 bp in the CMB isolates compared to that in the isolates from WAF (73 bp), TMB (242 bp), and TCB (275 bp) (**Figure 2**). The LD decay rate in CMB was slower than that in WAF, but faster than that in TCB and TMB.

**Figure 3:**
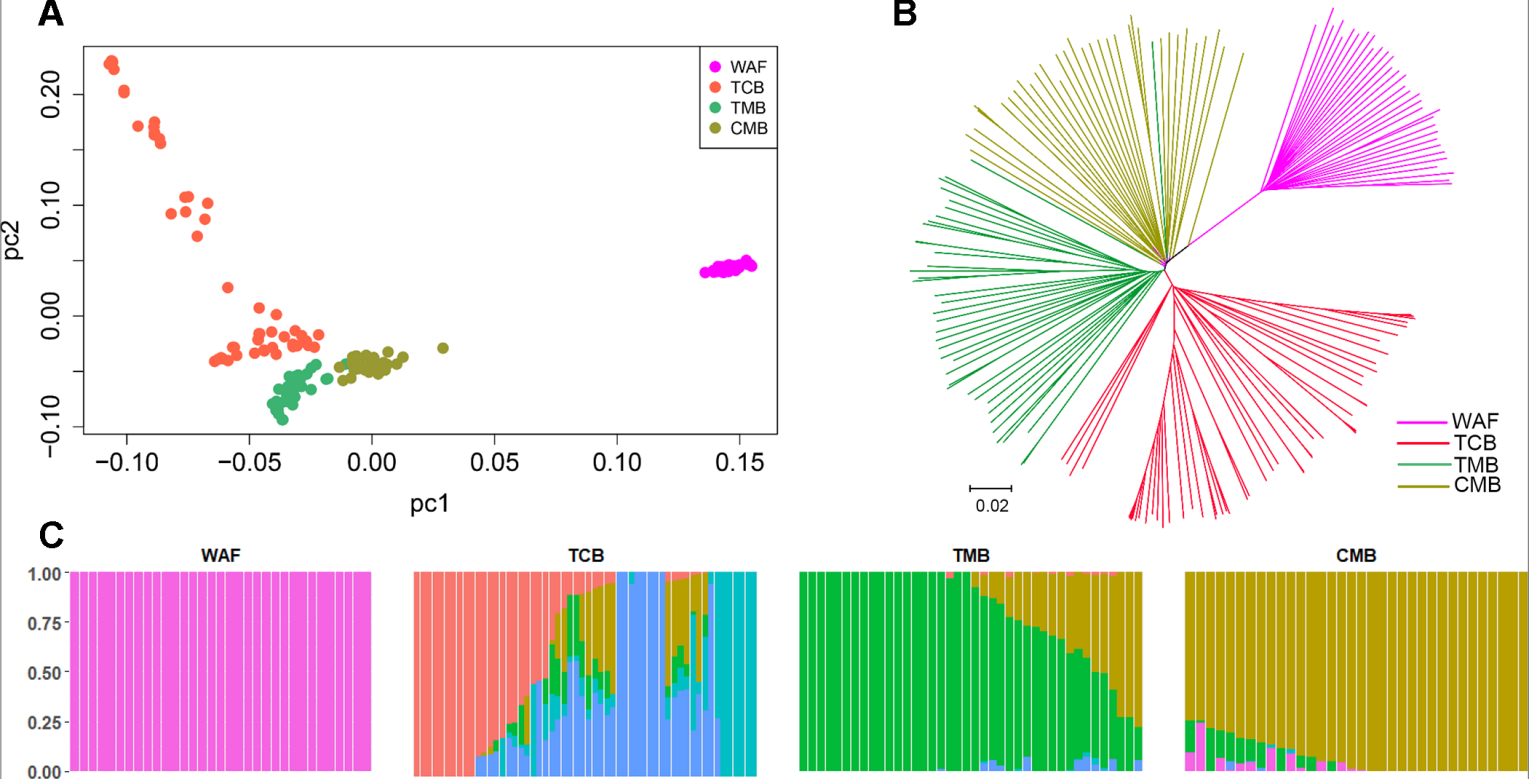
Population structure in the sample set analyzed. **(A**–**C)** Principal component analysis **(A)**, a neighbor-joining tree **(B)** and an admixture analysis **(C)**. Principal component analysis identified the four groups clearly. The neighbor-joining tree has four distinct branches separating four major components of population structure that correspond to the four geographical groups of sample; samples from WAF are separated from TCB, TMB, and CMB by long branches. Admixture analysis distinguished these four major components according to an optimized cluster value of *K* = 6, and multiple parasite subpopulations were found in TCB. WAF, West Africa; TCB, Thai–Cambodia border; TMB, Thai–Myanmar border; CMB, China–Myanmar border.

PCA and neighbor-joining and admixture analysis were performed to investigate major geographical divisions of population structures. The outcome from PCA distinguished clearly four major groups of the isolates, which are in accordance with their geographical origins (**Figure 3A**). We also performed PCA of the 34 isolates from CMB and found one major cluster with three minor clusters, which was consistent with a previous study (Wang et al., 2016) (**Supplementary Figure 3**). Similarly, the neighbor-joining analysis yielded a tree that is in accordance with the PCA outcome. The isolates from the four regions were clearly distinguished, and only one exception is that two isolates from TMB spread to the branch of CMB (**Figure 3B**). Meanwhile, the admixture analysis showed that six major components were differentiated according to the optimized cluster value of *K* = 6, and multiple parasite subpopulations were also found in TCB parasites (**Figure 3C**). There was gene flow among the isolates from Asia regions. In addition, the ancestral alleles in WAF isolates were identified in CMB but not in other Asia regions. As expected, the population clustering was consistent with fixation index (*F* *_ST_*) values (**Table 1**). *F* *_ST_* values of WAF isolates versus those from the three Asia regions ranged from 0.2 to 0.3, which suggested a great population differentiation between WAF and the Asia regions. Equally, the *F* *_ST_* values (*F* *_ST_* = 0.05) between CMB and TMB suggested a small population division.

**Table 1:** Pairwise differentiation between isolates from four regions, estimated using the *F* *_ST_* values.

Region	WAF	TCB	TMB	CMB
WAF	—	0.30	0.26	0.20
TCB	—	—	0.11	0.10
TMB	—	—	—	0.05
CMB	—	—	—	—

**Figure 4:**
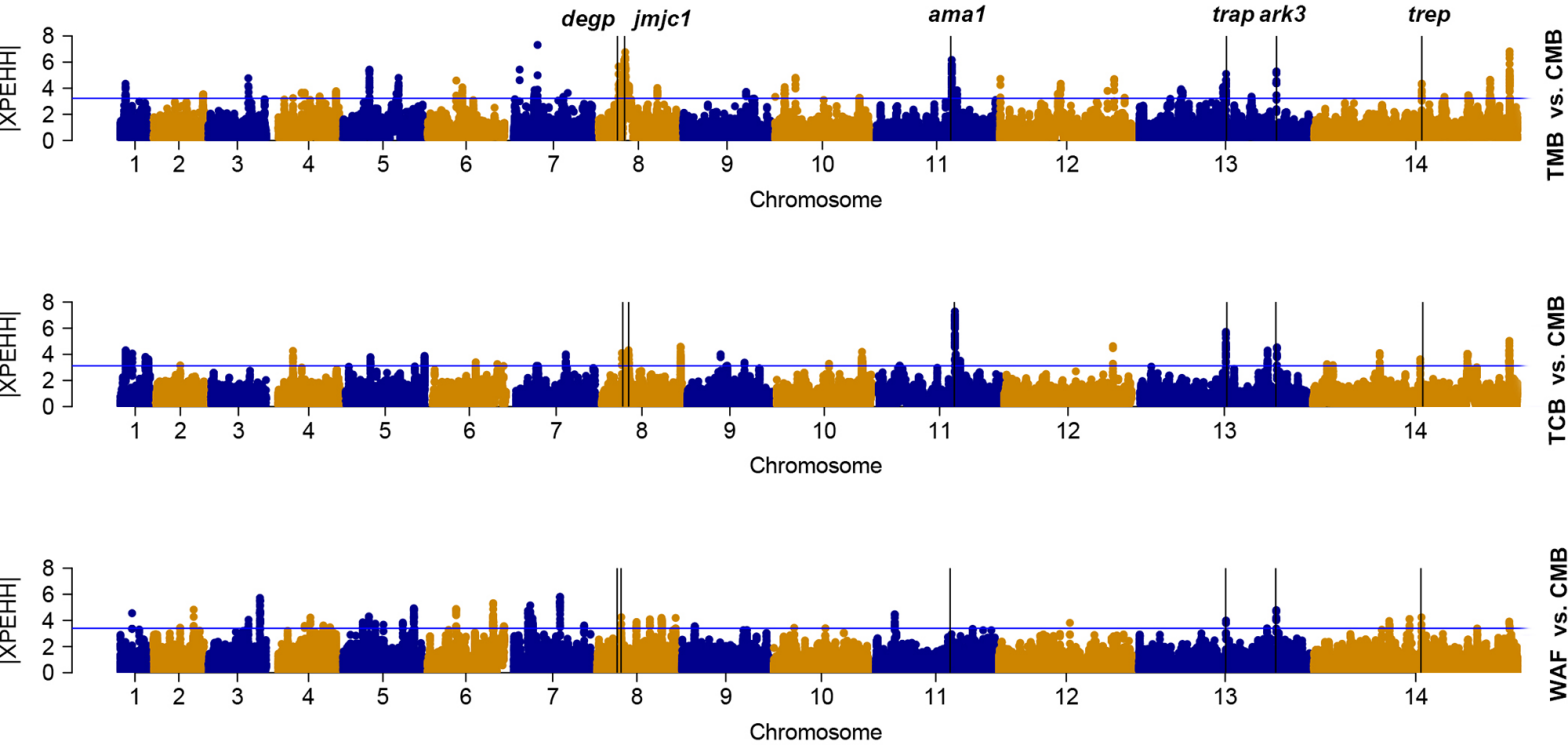
The top |XPEHH| hits for WAF, TCB, and TMB, using CMB as reference. *X* axis is chromosomes 1 to 14 in alternating colors; *y* axis is the value of |XPEHH|. The loci above the blue horizontal lines have the top 1% of |XPEHH| values; vertical lines represent the chromosome locations of three genes detected across the isolates from the other three regions (WAF, TCB, and TMB).

We used the cross-population extended-haplotype homozygosity (XP-EHH) to detect signals of positive selection locus that have reached fixation in the populations. We apply the top 1% expected distribution of XP-EHH score as a strong hits threshold. With CMB isolates as the reference population, signals of *trap*, *trep*, and *ark3* were identified directly across the isolates from the other three regions (**Figure 4**); signals such as *ruvb1*, *degp*, *jmjc1*, and *ama1* were detected in two Southeast Asian groups (**Supplementary Table 7**).

### Identification of a Set of SNPs for Tracing Origins of New Infections

We next attempted to find out a set of neutral SNPs to trace origins of the parasite infections. Top 600 most differential SNPs were obtained after pairwise comparison of the parasite SNPs from the four regions, of which 132 were neutral loci. We performed Stepwise Discriminant Analysis based on the neutral loci and identified 33 neutral SNPs (designated as “33 SNP barcode”; **Supplementary Table 8**) that can effectively distinguish the source of the isolates from the four regions; the coefficients of the 33 SNPs are listed in **Supplementary Table 9**. All the 163 isolates described above were analyzed on the 33-SNP barcode by retrospective retest and cross validation test, and the accuracy of tracing the source of the samples was 100% and 96.69%, respectively. To further validate the efficacy of the 33-SNP barcode, we obtained genome sequence data of additional 51 *P. falciparum* isolates, including 30 isolates with their genome sequence obtained from ENA (TCB, n = 10; TMB, n = 10; WAF, n = 10), 21 isolates with their genome sequence obtained through genome sequencing (CMB, n = 10; Hainan province of China, n = 1; Trat and Srisaket of Thailand, n = 8; Nigeria, n = 2). We show the sensitivity and specificity of the 33-SNP barcode in tracing the origins of the isolates from four regions in **Table 2** and **Supplementary Table 4**.

**Table 2:** Sensitivity and specificity of the 33–single-nucleotide polymorphism barcode in tracing the origins of the isolates from four regions.

Region	Sensitivity	Specificity
CMB	82%	95%
WAF	100%	100%
TCB	89%	97%
TMB	80%	97%

## Discussion

*P. falciparum* originated in Africa and spread to other continents along with human migration gradually formed new populations (Joy et al., 2003). In Southeast Asia with low malaria transmission, *P. falciparum* from Cambodia and Thailand form separate populations, potential barriers to migration were also detected along the CMB (Manske et al., 2012; Miotto et al., 2015; Shetty et al., 2019). Our previous study showed that the isolates from CMB clustered separately from those in TCB and TMB based on microsatellite diversity analysis (Ye et al., 2016). In this study, both the PCA and neighbor-joining tree analysis revealed that the parasites derived from CMB form a distinct population from other regions including TCB, TMB, and WAF. The admixture analysis showed that ancestral alleles from CMB isolates were found in both TMB and TCB, while the ancestral alleles from WAF isolates were found only in CMB, but not in both TMB and TCB.

The isolates of *P. falciparum* used for this study were mostly collected from the CMB. The isolates have been cultured *in vitro* for 2 to 4 weeks before genome sequencing, similar to the other studies using the cultured samples for the genome sequencing (Manske et al., 2012; Miotto et al., 2013). Although a long-term cultivation of malaria parasite may result in the clone loss and chromosomal deletions, we believe that such a short-term cultivation should have a limited impact on their genome diversity. Among the 43 isolates collected, six were estimated as mixed infections, which accounted for 14% of all the isolates in the Nabang–Lazan valley, which was similar to the previous report using the microsatellite analysis (Wei et al., 2015).

We showed that the average nucleotide diversity of *P. falciparum* isolates from CMB was higher than that from the parasites from TMB and TCB, but lower than the parasites from WAF. The *F* *_ST_* values from pairwise comparisons suggested that the isolates from CMB are more similar to those from TMB. In addition, rapid LD decay represents the high levels of recombination and outcrossing. We showed that the LD decay rate for the CMB parasites was faster than that for the parasites from Cambodia and Thailand, which was not consistent with the outcome previously reported using SNP array-based genome-wide genotyping (Wang et al., 2016).

We identified multiple genes that are likely under recent positive directional selection in *P. falciparum* isolates from CMB by computation of iHS, including those that encode important vaccine candidates and other membrane and surface proteins, which were previously observed in both Asian and African parasite populations (Mu et al., 2007; Ocholla et al., 2014; Samad et al., 2015). The deubiquitinating protease gene *pfubp1* was also detected (Borrmann et al., 2013), which may be associated with ART resistance. Selection signals at *pfcrt*, *pfdhfr*, and *pfdhps* were not detected in this study by iHS analysis. The explanation for this could be that the mutant alleles of the genes are already fixed in the local parasite population, e.g., *pfcrt*: K76T (88%), *pfdhfr*: S180N (100%), and *pfdhps*: K540E (79%) (**Supplementary Table 10**). We also identified several genes in the CMB parasites undergoing evolutionary interactions with the local human and mosquito hosts that were different from other regions. For example, *pfjmjc1*, a *jmjc* domain-containing histone lysine demethylases, plays a role in reversing histone lysine methylation, which is important to the parasite development and pathogenesis (Cui et al., 2008). *pfdegp* is a secretory multifunctional serine protease, which confers protection against thermal/oxidative stress (Sharma et al., 2014). The maximum level of *pfdegp* transcripts was observed in trophozoite and schizont stage (32 and 42 hpi), coinciding with the time when artemisinin was highly activated in the parasite (Wang et al., 2015). Since artemisinin acts through oxidative damage, *pfdegp* may be associated with drug- resistance. The *pfark3* gene encodes serine/threonine protein kinase, which contains 4,044 amino acids. The kinase domain sequence is highly similar to protein kinase A and calcium-dependent protein kinase (Anamika et al., 2005). It belongs to the aurora-related kinase (ARK) family of *P. falciparum*, and the function of this family of kinases may be related to the regulation of endocytosis and actin skeleton (Smythe and Ayscough, 2003; Cai et al., 2013). Its N-terminal and C-terminal regions are 33% and 45% homologous to a chloroquine resistance–associated protein of *Plasmodium yoelii*, respectively, implying that the mutation might be related to the drug resistant selection in this region.

Several attempts have been made to establish SNP barcode for defining the geographic origin of new infection with malaria parasite (Daniels et al., 2008; Campino et al., 2011; Preston et al., 2014). It is easier to distinguish parasite isolates from different continents than those from neighboring regions. So far, there are few molecular markers available to distinguish the parasite isolates within the Southeast Asia regions, where multiple drug-resistant parasites originated and spread. Neutral SNPs that disentangled the differential roles of natural selection are ideal genetic markers for tracing the origin and migration of the parasites. Here we identified 33 neutral SNPs to effectively classify the 163 samples from four different endemic regions and validated their accuracy using additional 51 samples. The 33 noncoding SNPs were identified as high-quality discriminant SNPs for use in differentiating parasites from different geographic areas. Because only two of the 33 SNPs are located in homopolymeric stretches of As and Ts, some methods such as MassARRAY platform (Manske et al., 2012), high-resolution melting analysis (Baniecki et al., 2015), and PCR-based Sanger sequencing can be used to call the SNPs. Therefore, these SNPs identified in this study should have practical operability in tracing the geographical origins of the isolates from CMB, TCB, and TMB.

## Data Availability Statement

Illumina sequencing reads from this study are available in the European Nucleotide Archive (PRJEB32255). The SNP data are available in the European Variation Archive (PRJEB34415).

## Ethics Statement

The studies involving human participants were reviewed and approved by Internal Review Board of Naval Medical University. The patients/participants provided their written informed consent to participate in this study.

## Author Contributions

RY, DZ, and WP conceived and designed this study. JW, XS, and HZ collected the samples. RY and YH performed the experiments. YT, RY, YZ, and DZ analyzed the data. RY, YT, and WP drafted the manuscript.

## Funding

This work was supported by grants from the National Natural Science Foundation of China (81601780 and 81220108019).

## Conflict of Interest

The authors declare that the research was conducted in the absence of any commercial or financial relationships that could be construed as a potential conflict of interest.
