# Supplementary material for: Genome-Wide Analysis of Genetic Diversity in Plasmodium falciparum Isolates From China–Myanmar Border
Source: Front Genet. 2019 Oct 29;10:1065. doi: 10.3389/fgene.2019.01065 (PMC6830057; doi:10.3389/fgene.2019.01065)
Supplement: Supplementary file 6 [file Table_6.docx]

**Supplementary Table 6**. 32 Genes with at least 2 SNPs in the top 1% of *|iHS|* values in China-Myanmar border, with median *|iHS|* per gene.

| **Chr** | **Gene ID** | **iHS** | **SNPs** | **ANNOTATION** |
| --- | --- | --- | --- | --- |
| 1 | PF3D7_0104100 | 3.33 | 3 | Conserved Plasmodium membrane protein, unknown function |
| 1 | PF3D7_0104300 | 3.63 | 2 | Ubiquitin carboxyl-terminal hydrolase_1 |
| 1 | PF3D7_0113800 | 3.69 | 5 | DBL containing protein, unknown function |
| 2 | PF3D7_0214300 | 3.93 | 2 | conserved Plasmodium protein, unknown function |
| 3 | PF3D7_0302200 | 3.76 | 2 | cytoadherence linked asexual protein 3.2 |
| 4 | PF3D7_0405300 | 3.29 | 2 | 6-cysteine protein (LISP2) |
| 4 | PF3D7_0420000 | 3.37 | 2 | zinc finger protein, putative |
| 4 | PF3D7_0424400 | 3.50 | 17 | surface-associated interspersed protein 4.2 |
| 4 | PF3D7_0424800 | 5.60 | 26 | Plasmodium exported protein (PHISTb), unknown function |
| 5 | PF3D7_0506700 | 3.62 | 2 | GTPase-activating protein, putative |
| 6 | PF3D7_0609700 | 3.59 | 2 | conserved Plasmodium protein, unknown function |
| 6 | PF3D7_0630400 | 4.37 | 2 | conserved Plasmodium protein, unknown function |
| 7 | PF3D7_0710000 | 3.32 | 2 | conserved Plasmodium protein, unknown function |
| 7 | PF3D7_0710200 | 3.50 | 2 | conserved Plasmodium protein, unknown function |
| 7 | PF3D7_0714600 | 3.24 | 2 | conserved Plasmodium protein, unknown function |
| 8 | PF3D7_0823300 | 3.96 | 2 | histone acetyltransferase GCN5 (GCN5) |
| 8 | PF3D7_0826000 | 3.76 | 2 | conserved Plasmodium protein, unknown function |
| 8 | PF3D7_0830800 | 4.45 | 9 | surface-associated interspersed protein 8.2 (SURFIN 8.2) |
| 10 | PF3D7_1032700 | 3.50 | 2 | conserved Plasmodium protein, unknown function |
| 10 | PF3D7_1038400 | 3.49 | 2 | gametocyte-specific protein (Pf11-1) |
| 11 | PF3D7_1133400 | 3.72 | 8 | apical membrane antigen 1 (AMA1) |
| 11 | PF3D7_1136600 | 3.53 | 2 | conserved Plasmodium protein, unknown function |
| 11 | PF3D7_1148700 | 3.59 | 4 | Plasmodium exported protein (PHISTc),unknown function |
| 12 | PF3D7_1203300 | 3.54 | 2 | conserved Plasmodium protein, unknown function |
| 12 | PF3D7_1216600 | 4.10 | 2 | cell traversal protein for ookinetes and sporozoites (CelTOS) |
| 13 | PF3D7_1328200 | 3.61 | 4 | conserved Plasmodium protein, unknown function |
| 13 | PF3D7_1335900 | 4.22 | 2 | thrombospondin-related anonymous protein (TRAP) |
| 13 | PF3D7_1356800 | 5.29 | 3 | serine/threonine protein kinase, putative (ARK3) |
| 14 | PF3D7_1428400 | 3.57 | 2 | WD and tetratricopeptide repeats protein1, putative (WDTC1) |
| 14 | PF3D7_1429800 | 4.31 | 2 | coatamer beta subunit, putative |
| 14 | PF3D7_1475800 | 3.78 | 7 | conserved Plasmodium protein, unknown function |
| 14 | PF3D7_1477600 | 3.51 | 2 | surface-associated interspersed protein 14.1 |
