# Supplementary material for: Genome-Wide Analysis of Genetic Diversity in Plasmodium falciparum Isolates From China–Myanmar Border
Source: Front Genet. 2019 Oct 29;10:1065. doi: 10.3389/fgene.2019.01065 (PMC6830057; doi:10.3389/fgene.2019.01065)
Supplement: Supplementary file 7 [file Table_7.docx]

**Supplementary Table 7.** Genes with SNPs in the top 1% of |XP-EHH| values in each region with China-Myanmar border as the reference population. Median |XP-EHH| values per gene are shown for the 90 genes with at least 2 SNPs.

| **Chr** | **Gene ID** | **Gene**  **name** | **TM**  **(XP-EHH)** | **TC**  **(XP-EHH)** | **WF**  **(XP-EHH)** | **Total SNPs** |
| --- | --- | --- | --- | --- | --- | --- |
| 1 | PF3D7_0104100 |  | 3.50 | 3.52 | - | 53 |
| 1 | PF3D7_0106700 | *AARP2* | - | 3.53 | 3.36 | 6 |
| 1 | PF3D7_0113000 | *GARP* | - | 3.35 | - | 2 |
| 1 | PF3D7_0113600 | *SURFIN 1.2* | - | 3.36 | - | 2 |
| 1 | PF3D7_0113800 |  | - | 3.37 | - | 26 |
| 1 | PF3D7_0110500 |  | - | - | 3.20 | 2 |
| 2 | PF3D7_0212100 |  | - | - | 3.39 | 5 |
| 2 | PF3D7_0216800 |  | - | - | 3.57 | 6 |
| 3 | PF3D7_0315300 |  | - | - | 3.16 | 2 |
| 3 | PF3D7_0317300 |  | 4.12 | - | 3.50 | 11 |
| 3 | PF3D7_0321200 | *ALG7* | - | - | 4.65 | 19 |
| 4 | PF3D7_0406500 |  | - | - | 3.11 | 2 |
| 4 | PF3D7_0408700 | *PLP1* | - | 3.69 | - | 6 |
| 4 | PF3D7_0415800 |  | - | - | 3.81 | 4 |
| 4 | PF3D7_0419900 |  | - | - | 3.08 | 2 |
| 4 | PF3D7_0420000 |  | - | - | 3.57 | 3 |
| 4 | PF3D7_0422200 |  | - | - | 3.28 | 3 |
| 4 | PF3D7_0424400 | *SURFIN 4.2* | 3.49 | - | - | 25 |
| 5 | PF3D7_0511300 |  | - | - | 4.03 | 5 |
| 5 | PF3D7_0511400 |  | 4.76 | 3.40 | - | 18 |
| 5 | PF3D7_0513600 |  | - | - | 3.63 | 3 |
| 5 | PF3D7_0516800 | *ApiAP2* | - | - | 3.36 | 2 |
| 5 | PF3D7_0522600 |  | 3.86 | - | - | 2 |
| 5 | PF3D7_0524300 |  | - | - | 3.66 | 5 |
| 5 | PF3D7_0522700 | *SufA* | 3.85 | - | - | 2 |
| 5 | PF3D7_0528900 |  | - | - | 3.91 | 7 |
| 5 | PF3D7_0529000 |  | - | - | 3.23 | 5 |
| 5 | PF3D7_0529200 |  | - | - | 3.18 | 4 |
| 6 | PF3D7_0609600 |  | - | - | 3.15 | 2 |
| 6 | PF3D7_0612800 | *P12p* | 4.01 | - | 4.40 | 11 |
| 6 | PF3D7_0615100 |  | 3.93 | - | - | 2 |
| 6 | PF3D7_0627700 |  | - | - | 3.66 | 3 |
| 6 | PF3D7_0627800 | *ACS* | - | - | 3.56 | 16 |
| 6 | PF3D7_0630300 |  | - | - | 3.05 | 2 |
| 6 | PF3D7_0630600 |  | - | - | 3.51 | 5 |
| 7 | PF3D7_0708200 |  | - | - | 3.79 | 9 |
| 7 | PF3D7_0704800 |  | 5.02 | - | - | 2 |
| 7 | PF3D7_0709100 | *Cg1* | - | - | 3.48 | 24 |
| 7 | PF3D7_0709200 | *GLP3* | - | - | 3.10 | 2 |
| 7 | PF3D7_0721600 |  | - | 3.38 | - | 3 |
| 7 | PF3D7_0710000 |  | - | - | 3.38 | 19 |
| 7 | PF3D7_0720400 |  | - | - | 3.55 | 2 |
| 7 | PF3D7_0729700 |  | - | - | 3.22 | 7 |
| 8 | PF3D7_0807700 | *DegP* | 5.08 | 3.68 | - | 6 |
| 8 | PF3D7_0808100 |  | 3.72 | - | - | 5 |
| 8 | PF3D7_0808200 |  | 4.06 | - | - | 4 |
| 8 | PF3D7_0809200 | *pfa55-14* | 4.43 | - | - |  |
| 8 | PF3D7_0809400 |  | - | 3.23 | - | 5 |
| 8 | PF3D7_0809600 |  | 4.39 | 3.72 | - | 70 |
| 8 | PF3D7_0809700 | *RUVB1* | 5.25 | 3.62 | - | 22 |
| 8 | PF3D7_0809800 |  | 5.25 | 3.89 | - | 22 |
| 8 | PF3D7_0809900 | *JmjC1* | 4.83 | 3.31 | - | 13 |
| 8 | PF3D7_0810600 |  | 3.65 | - | - | 4 |
| 8 | PF3D7_0810800 | *PPPK-DHPS* | 3.70 | - | - | 5 |
| 8 | PF3D7_0811200 | *EMC1* | 4.01 | - | - | 5 |
| 8 | PF3D7_0814700 |  | - | - | 3.45 | 6 |
| 8 | PF3D7_0820300 |  | - | - | 3.59 | 6 |
| 8 | PF3D7_0822900 |  | 3.74 | - | - | 7 |
| 8 | PF3D7_0825700 |  | - | - | 3.23 | 3 |
| 8 | PF3D7_0825800 |  | - | - | 3.78 | 4 |
| 8 | PF3D7_0826000 |  | - | - | 3.43 | 8 |
| 8 | PF3D7_0831100 | *SURFIN 8.1* | - | - | 3.38 | 5 |
| 8 | PF3D7_0830800 | *SURFIN 8.2* | - | 3.63 | - | 44 |
| 9 | PF3D7_0914000 |  | - | 3.89 | - | 2 |
| 9 | PF3D7_0905700.3 |  | - | - | 3.39 | 8 |
| 9 | PF3D7_0926500 |  | 3.62 | - | - | 4 |
| 9 | PF3D7_0935800 | *CLAG9* | - | - | 3.03 | 2 |
| 10 | PF3D7_1004600 |  | 3.60 | - | - | 12 |
| 10 | PF3D7_1009600 |  | 4.68 | - | - | 6 |
| 10 | PF3D7_1028500 |  | - | - | 3.04 | 2 |
| 11 | PF3D7_1110300 |  | - | - | 3.91 | 6 |
| 11 | PF3D7_1140900 |  | - | - | 3.27 | 4 |
| 11 | PF3D7_1133400 | *AMA1* | 4.67 | 5.53 | - | 64 |
| 11 | PF3D7_1135200 |  | 3.53 | 3.21 | - | 4 |
| 12 | PF3D7_1223600 |  | 3.53 | - | - | 2 |
| 12 | PF3D7_1244200 | *TFB2* | 4.29 | - | - | 5 |
| 12 | PF3D7_1241200 |  | - | 4.48 | - | 3 |
| 13 | PF3D7_1318300 |  | 3.82 | - | - | 5 |
| 13 | PF3D7_1318900 |  | 3.62 | - | - | 5 |
| 13 | PF3D7_1335100 | *MSP7* | 3.97 | - | - | 5 |
| 13 | PF3D7_1335900 | *TRAP* | 3.74 | 4.42 | 3.04 | 55 |
| 13 | PF3D7_1352900 |  | - | 3.51 | 3.13 | 4 |
| 13 | PF3D7_1356800 | *ARK3* | 4.72 | 3.92 | 4.04 | 20 |
| 14 | PF3D7_1431200 |  | - | - | 3.61 | 4 |
| 14 | PF3D7_1442600 | *TREP* | 3.84 | 3.49 | 3.52 | 11 |
| 14 | PF3D7_1464500 |  | - | - | 3.26 | 4 |
| 14 | PF3D7_1460500 |  | 3.45 | 3.87 | - | 11 |
| 14 | PF3D7_1468500 | *DER1-1* | 4.59 | - | - | 3 |
| 14 | PF3D7_1475800 |  | 4.67 | 3.69 | 3.83 | 56 |
| 14 | PF3D7_1475900 |  | - | - | 3.46 |  |
