# Supplementary material for: Genome-Wide Analysis of Genetic Diversity in Plasmodium falciparum Isolates From China–Myanmar Border
Source: Front Genet. 2019 Oct 29;10:1065. doi: 10.3389/fgene.2019.01065 (PMC6830057; doi:10.3389/fgene.2019.01065)
Supplement: Supplementary file 11 [file DataSheet_1.pdf]

Supplementary Material

Supplementary Figures

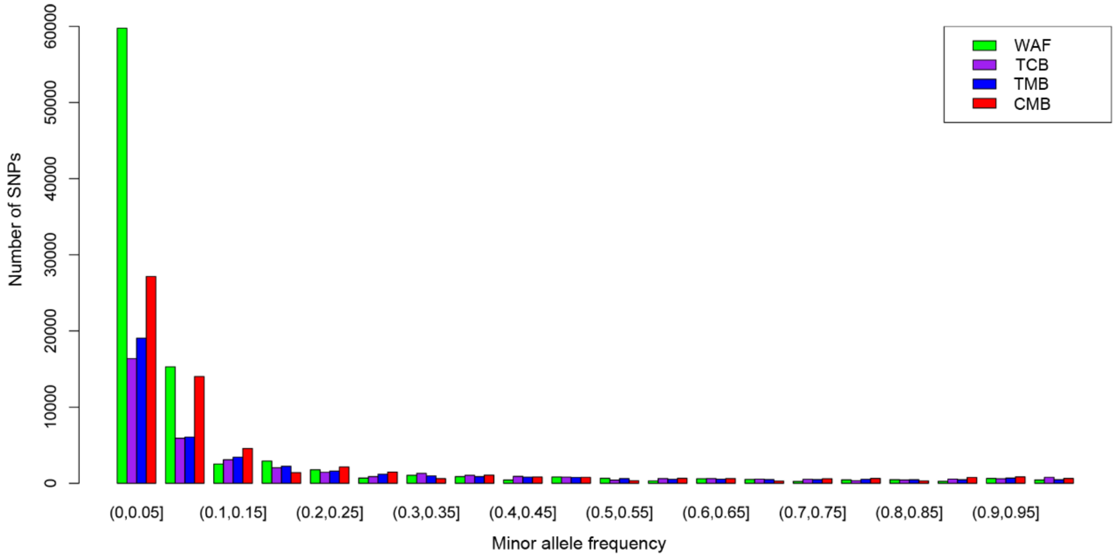

Supplementary Figure 1. Minor allele frequency(MAF) distribution of the SNPs in samples from four regions: China-Myanmar border(CMB), Thailand-Myanmar border(TMB), Thailand-Cambodia border(TCB), west Africa(WAF).

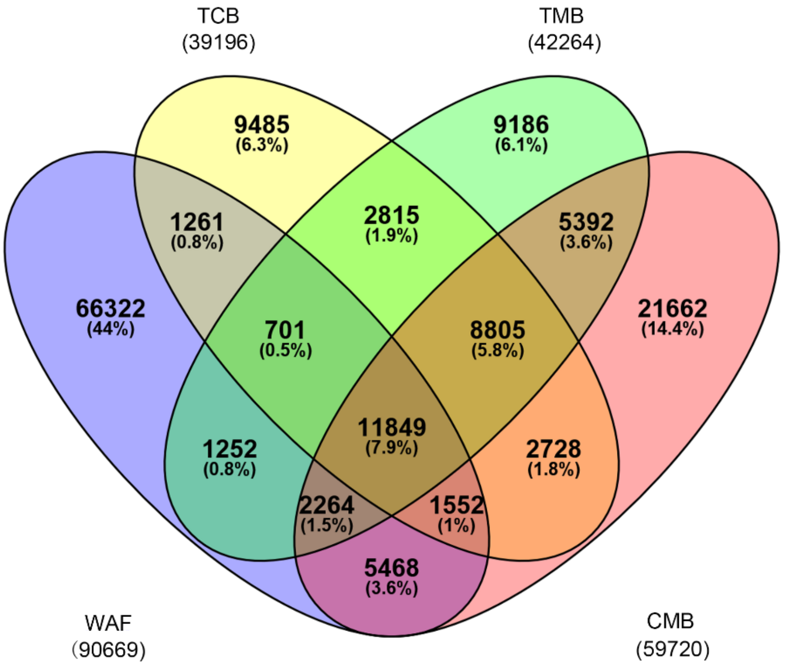

Supplementary Figure 2 Venn diagram showing the number of SNPs shared by samples from four epidemic areas. West Africa (WAF, SNP=90669), Thai-Cambodia border (TCB, SNP=39196), Thai-

Myanmar border (TMB, SNP=42246), China-Myanmar border (CMB, SNP=59720). Private SNPs: WAF(n=66322), TCB(n=9485), TMB(n=9186), CMB(n=21662). Overall, 11849 SNPs were shared by the four populations. CMB and TMB shared the largest number of SNPs (n= 28,310), followed by CMB and TCB(n=24934). Each oval represented one region.

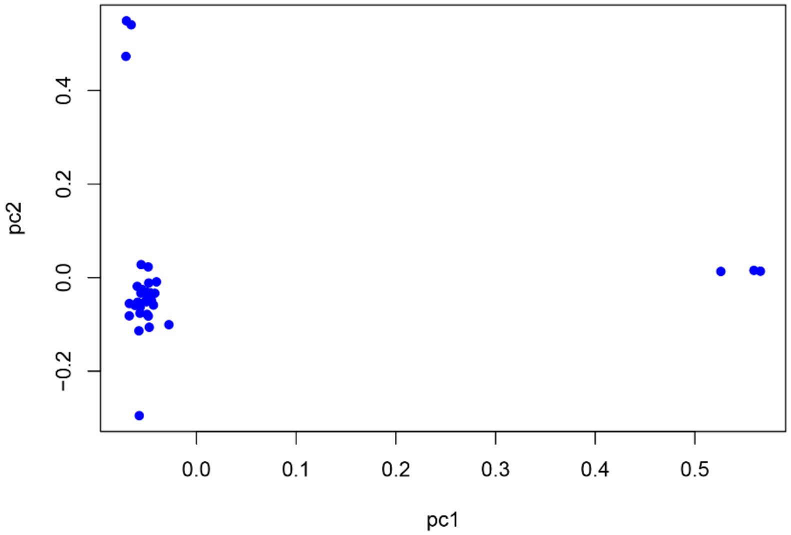

Supplementary Figure 3. PCA plots of the 34 isolates from CMB.

Supplementary Tables

Supplementary Table 1. Summary of samples with high-quality genomic data (Supplementary Table 1.xlsx).

Supplementary Table 2. Summary of samples from TMB, TCB and WAF downloaded from European Nucleotide Archive(Supplementary Table 2.xlsx)

Supplementary Table 3. The *P. falciparum* subtelomeric regions. (Supplementary Table 3.docx)

Supplementary Table 4. The geographic origin of 51 samples tested by the 33 SNPs barcode. (Supplementary Table 4.xlsx)

Supplementary Table 5. 158 genes with at least 5 SNPs with Tajima D values >1 in China-Myanmar border (Supplementary Table 5.xlsx).

Supplementary Table 6. 32 Genes with at least 2 SNPs in the top 1% of |*iHS*| values in China-Myanmar border, with median |*iHS*| per gene. (Supplementary Table 6.docx)

Supplementary Table 7. Genes with SNPs in the top 1% of |XP-EHH| values in each region with China-Myanmar border as the reference population. Median |XP-EHH| values per gene are shown for the 90 genes with at least 2 SNPs. (Supplementary Table 7.docx)

Supplementary Table 8. Genomic information for 33 SNPs, including the chromosome position, Gene ID where the SNP is located, SNP type (Synonymous, Intron or Intergenic), and the reference and alternate alleles. (Supplementary Table 8.xlsx)

Supplementary Table 9. Coefficients of the 33 SNPs for the Stepwise Discriminant Analysis (Supplementary Table 9.xlsx)

Supplementary Table 10. Frequency of alleles conferring drug resistance in four regions. (Supplementary Table 10.xlsx)
